# Characterization of insulin cross-seeding: the underlying mechanism reveals seeding and denaturant-induced insulin fibrillation proceeds through structurally similar intermediates†

**DOI:** 10.1039/d0ra05414c

**Published:** 2020-08-13

**Authors:** Mohsen Akbarian, Maryam Kianpour, Reza Yousefi, Ali Akbar Moosavi-Movahedi

**Affiliations:** Protein Chemistry Laboratory (PCL), Department of Biology, College of Sciences, Shiraz University Shiraz Iran; Institute of Biochemistry and Biophysics (IBB), The University of Tehran Tehran Iran ryousefi@shirazu.ac.ir +98 71 32280916 +98 71 36137617

## Abstract

Insulin rapidly fibrillates in the presence of amyloid seeds from different sources. To address its cross-reactivity we chose the seeds of seven model proteins and peptides along with the seeds of insulin itself. Model candidates were selected/designed according to their size, amino acid sequence, and hydrophobicity. We found while some seeds provided catalytic ends for inducing the formation of non-native insulin conformers and increase fibrillation, others attenuated insulin fibrillation kinetics. We also observed competition between the intermediate insulin conformers which formed with urea and amyloid seeds in entering the fibrillogenic pathway. Simultaneous incubation of insulin with urea and amyloid seeds resulted in the formation of nearly similar insulin intermediate conformers which synergistically enhance insulin fibrillation kinetics. Given these results, it is highly likely that, structurally, there is a specific intermediate in different pathways of insulin fibrillation that governs fibrillation kinetics and morphology of the final mature fibril. Overall, this study provides a novel mechanistic insight into insulin fibrillation and gives new information on how seeds of different proteins are capable of altering insulin fibrillation kinetics and morphology. This report, for the first time, tries to answer an important question that why fibrillation of insulin is either accelerated or attenuated in the presence of amyloid fibril seeds from different sources.

## Abstract 2

Native insulins in the presence of low urea concentrations or seeds with low hydrophobicity form ordered aggregates (amyloid fibrils), while high urea concentrations or the seeds with high level of hydrophobicity can induce the amorphous aggregation.

## Graphical abstract

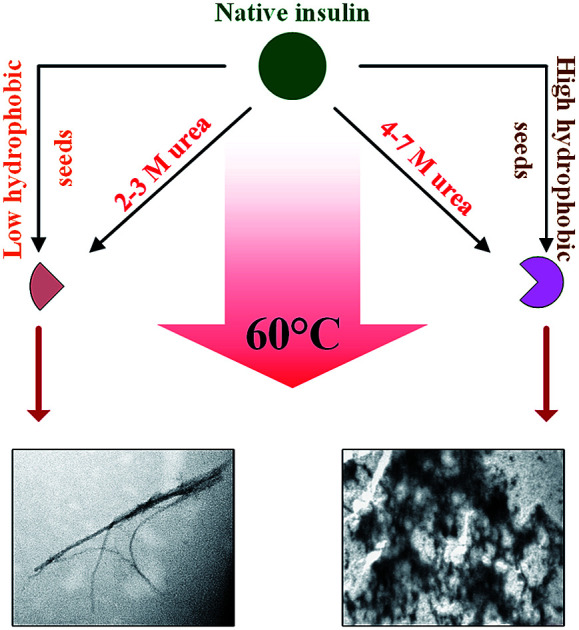

## Introduction

Fibrillary deposits cause serious problems during commercial insulin production, in implantable insulin pumps and some diabetic patients.^1–3^ Despite numerous efforts, elucidation of the exact mechanism of protein fibrillation has eluded the investigators so far. Amyloid fibrillation is a process in which native soluble protein becomes misfolded into insoluble fibril, rich in cross β-sheet structure.^4–6^ Beginning in the lag-phase, partially unfolded proteins convert into oligomers and then protofilaments both of which are mostly non-detectable by the current methods. Thereafter, the fibril population increases rapidly during the elongation phase.^7^ The elongation issue gets more complicated when seeding molecules or foreign surfaces influence the kinetics of protein fibrillation.^8,9^ In our previous study, it was found that hydrophobicity and electrostatic forces on the external surfaces have distinct effects on insulin fibrillation.^10^ Also, during fibrillation of amyloid precursor proteins, β2-microglobulin, and insulin the lag-phase is reduced in the presence of pre-existing fibril seeds,^11–13^ however, no sufficient description has been presented about elongation kinetics. Although the seeding- or secondary nucleation-induced fibrillation is an ambiguous phenomenon, it becomes even more complicated when the kinetics of fibrillation is affected through cross-reactivity between seeds originating from a different protein. Many efforts have been made to elucidate the cross-reactivity between the amyloidogenic peptides and proteins.^11,14^ In general, it is accepted that the added seeds will provide catalytic end(s), accelerating the rate of protein fibrillation.^14,15^ By considering this assumption, one may speculate that the kinetic of fibrillation is always speeded up in the presence of foreign protein seeds. However, it has been recently indicated that the amyloid seed of pro-islet amyloid polypeptide (proIAPP) is capable of inhibiting amyloid formation by α-synuclein.^11^ This then indicates cross-seeds might influence the structure of intact monomers of fibrillating protein. Generally, on the bases of cases examined yet, the rate of protein fibril formation increases upon the addition of its seed (secondary nucleation).^11,14,16,17^ A study on cross-interaction between β-amyloid (Aβ) and IAPP suggested that identity and similarity between the two protein sequences could affect the rate of their fibrillation. But it is not always the case. For example, seeds of huntingtin are capable to efficiently fibrillate the RNA-binding protein (TIA-1) although the two proteins have a low sequence similarity.^9^ Similarly, it was reported that fibrillation of insulin is significantly reduced in the presence of the seed of its B-chain^16^ at neutral pH. Likewise, it has been shown that amyloid fibrils from different proteins with low sequence similarity exhibited structural differences that control the seeding efficiencies.^18^ Yet another study suggested a relationship between the number of amino acids in the monomeric subunit and the size of protein fibril.^19^ It thus follows that the interactions between soluble proteins and pre-formed seeds at the molecular resolution are complex, elusive, and more studies are needed in understanding this process.

In addition to the efforts made so far in the secondary-seeding/cross-seeding experiments, our current knowledge about insulin fibrillation also comes from the study of chaotropic additives such as urea. Under monomeric condition and in the presence of low urea concentration, it has been shown that the rate of insulin fibrillation decreases, while the rate is significantly faster at the higher levels of this denaturing agent indicating the presence of several intermediate conformers with different susceptibilities toward entering on- or off-fibrillation pathway.^20^ It, therefore, becomes interesting to compare different scenarios. If seeding does affect the kinetics *i.e.*, accelerates or inhibits the rate of insulin fibrillation then it becomes compelling to explore if seeds induce structural modifications in the insulin molecules akin to chatropes and any other similarities in the mechanism thereof. To address these questions and to characterize the cross-seeding effect on the fibrillation of human recombinant insulin, herein, we used seven model proteins and peptides that vary based on their size, sequence, and hydrophobicity. The model proteins and peptides were human αB-crystallin (αB-Cry), human αA-crystallin (αA-Cry), N-terminal domain of αB-Cry (αB-NTD), a fusion protein of αB-Cry and insulin A-chain (αB-AC), the fusion protein of αB-Cry and insulin B-chain (αB-BC), insulin A-chain, and insulin B-chain. The seeds of intact human insulin served as a control to compare the results. Using several biophysical methods, *in vitro*, our data suggested that while the seed of αB-AC, insulin A- and B-chain and αB-Cry provided catalytic ends for accelerating insulin fibrillation, those of αA-Cry, αB-BC and αB-NTD were capable of inhibiting insulin fibrillation. Investigating the insulin fibrillation in the presence of insulin seed (secondary nucleation) revealed that the reaction rate was also significantly augmented. There was a similarity in the structural modification induced in insulin by seed type or denaturant. Overall, this study provides mechanistic insight into insulin fibrillation and how seeds from different proteins can alter the nature and kinetics of insulin aggregation. These studies will help us better understand the seed-induced insulin fibrillation process at the molecular level. The results will also help in the better formulation of this antidiabetic hormone.

## Materials and methods

### Materials

Bis-1-anilino-8-naphthalene sulfonate (bis-ANS), thioflavin T (ThT), urea, and other chemicals were purchased from Sigma.

## Methods

### Preparation of the proteins and peptides

In the current study, the chain combination method was used for producing recombinant human insulin.^21^ In brief, the plasmids (pET28b+) containing αB-BC (αB-Cry + B-chain) and αB-AC (αB-Cry + A-chain) fusion genes were constructed. Isopropyl β-d-1-thiogalactopyranoside (IPTG)-mediated expression of the fusion genes were done in *Escherichia coli* BL21 (DE3) pLysS cells (Invitrogen, Carlsbad, CA). The produced fusion proteins were purified by Ni-NTA (for αB-AC) and diethylaminoethyl (DEAE) anion exchange chromatography (for αB-BC). Owing to the presence of methionine residue between insulin chains and the fusion partner (αB-Cry), CNBr-mediated cleavage strategy was used for detaching the peptides from αB-Cry. For masking thiol groups of cysteine residues located in the chains of insulin sulfitolysis experiment was done, then due to the significant difference between sizes of fusion partner and the peptides, G50-Sephadex (Merck, Darmstadt, Germany) gel filtration was performed for separation/purification of the A- and B-chain of human insulin. After combination of these two chains (see ref. 21), natively folded insulin was obtained using phenyl Sepharose hydrophobic column (GE Healthcare, Chicago IL). To produce a mutant form of αB-Cry, Quick-change site-directed mutagenesis kit (Stratagene, La Jolla, CA) was used.^22^ As a hydrophobic part, the N-terminal domain of αB-Cry (αB-NTD) was obtained by CNBr cleavage at methionine 68 of wild type αB-Cry. Purification of the produced NTD was done by Sephadex G25 (90 × 1 cm).

### Kinetics of monomeric insulin fibrillation

A solution of acetic acid (20%, pH 2.0) containing 50 mM NaCl in varying concentrations of urea (at 60 °C) was selected as the fibrillogenic condition.^20^ The insulin sample (2 mg mL^−1^) was freshly prepared immediately before the experiments using a molar extinction coefficient of 1.08 at 276 nm for 1.0 mg mL^−1^.^4^ The growth of amyloid fibrils was monitored by measuring ThT fluorescence intensity at 484 nm. To perform this experiment, 10 μM ThT was added to each protein solution and then incubated in the black 96-well plate with the final volume of 100 μL. The fluorescence intensity at 482 nm was plotted against time, and the kinetic profiles were analyzed by curve fitting using GraphPad Prism V7.0 (GraphPad Software, Inc La Jolla, California, USA). For each sample, three replicates were measured to reduce well to well variation.

### Circular dichroism (CD) assessment

The circular dichroism (CD) spectra were taken on a Jasco J-810 spectropolarimeter using a cell with a 0.1 mm optical path length at 60 °C. The measurements were done in acetic acid (20%, pH 2.0) containing 50 mM NaCl and various urea concentrations ranging from 0.5 to 7.0 M. In each experiment, the concentration of insulin was set at 2.0 mg mL^−1^. To study the reversibility of the induced secondary structures under fibrillogenic condition, a serial dilution of urea concentrations was applied. Therefore, the protein solutions were individually dissolved with 7.0–1.0 M urea. After 15 min incubation, the solutions were diluted to a final concentration of 2.0 mg mL^−1^ for insulin and desired concentration of urea (6.0, 5.0, 4.0, 3.0, 2.5, 2.0, 1.5, 1.0 and 0.5 M). To analyze the α-helix transition during each step, molar ellipticities at 209 nm were plotted *versus* ascending and descending urea levels.

### Fourier transform infrared (FTIR) spectroscopy

At the end of fibrillation, for analyzing the secondary structure of fibrils, 100 μL of the aliquots (2.0 mg mL^−1^) were taken after 6.0 h of the incubation under fibrillogenic conditions. The samples were then centrifuged, separately. The generated fibrils were also centrifuged (20 min, 13 000 g at ambient temperature) and washed with D_2_O to exchange hydrogens and buffers by deuterons.^23,24^ This procedure was repeated at least three times for each sample. Finally, the fibrils were spread uniformly on the surface of an attenuated total reflectance (ATR) diamond cell. ATR-Fourier transform infrared (ATR-FTIR) spectra were recorded on a Tensor II instrument (Bruker, Germany) from 1700 to 1600 cm^−1^ (amide I), using a resolution of 4.0 cm^−1^ and an accumulation of 256 scans. Curve fitting of raw data and deconvolution analyses with Gaussian function were carried out by Origin 2018b software using the positions of five peaks. These positions have been previously used for the analysis of protein amyloid: 1600–1615 cm^−1^ (β-sheet, extended chain, side chain), 1615–1630 cm^−1^ (β-sheet), 1630–1650 cm^−1^ (α-helix), 1650–1690 cm^−1^ (turn, disordered), 1690–1700 cm^−1^ (antiparallel β-sheet) and random coil (1648 cm^−1^).^24^

### Transmission electron microscopic analyses

A 5-fold dilute solution of the fibrils in each sample was deposited on the formvar carbon-coated copper grids and negatively stained with 1.0% aqueous uranyl acetate.^25^ The specimens were examined at 100 kV excitation voltages, with a Philips CM10 transmission electron microscope (Philips, The Netherlands).

### Fluorescence microscopy measurements

At the end of fibrillation, to find out the fiber cross-connections and/or lateral interactions between them (bundles of the fibrils), the generated fibrils were mixed with ThT at the final concentration of 20 μM. Then, 50 μL of the samples were spread homogeneously onto a microscopic slide and incubated for 30 min at a dark place. Using excitation/emission filters (469/525 nm), the fluorescence images were obtained.

### The seeding experiments

In the present work, 2.0 mg mL^−1^ solution of the fibrillated proteins and peptides were collected by centrifuging the specimen (13 000*g* for 20 min at ambient temperature). The pellet of fibrils was dialyzed in double-distilled water and gently sonicated.^26^ After that, lyophilizing was done and residual fibril samples were weighted. Based on the images taken from the transmission electron microscope, the seeds were short, 40 nm. A 5% (w/w) of the seeds/insulin was prepared to study the kinetics of fibrillation.^14,16,26^ The defined fibrillogenic condition and the desired urea concentration were considered for the seeding experiments. Monitoring of the fibrillation was done as described before.

### Measuring the protein concentration

The concentrations of recombinant human insulin, insulin A-chain, insulin B-chain and the fusion proteins (αB-AC and αB-BC) were estimated based on previous publications.^6,21,25^ Also, following previous literature,^27^ αA-Cry concentration was determined by UV absorption at 280 nm (an extinction coefficient of 0.72, mL mg^−1^ cm^−1^, for 1.0 mg mL^−1^).

### Gel electrophoresis experiment

The purity of the proteins and peptides were analyzed by reducing SDS-PAGE (gel 18%). The gel stained using Coomassie Brilliant Blue (CBB) method.^28^

### Statistical analyses

The data were analyzed in GraphPad Prism and presented as mean ± S.E.M. The significance was statistically analyzed by one-way ANOVA using SigmaPlot 12.0 software. The statistical significance among the groups was determined using analyses of variance, and *P* < 0.05 was considered significant.^21,27^

## Results

### Designing seeding proteins and peptides

**Scheme 1:**
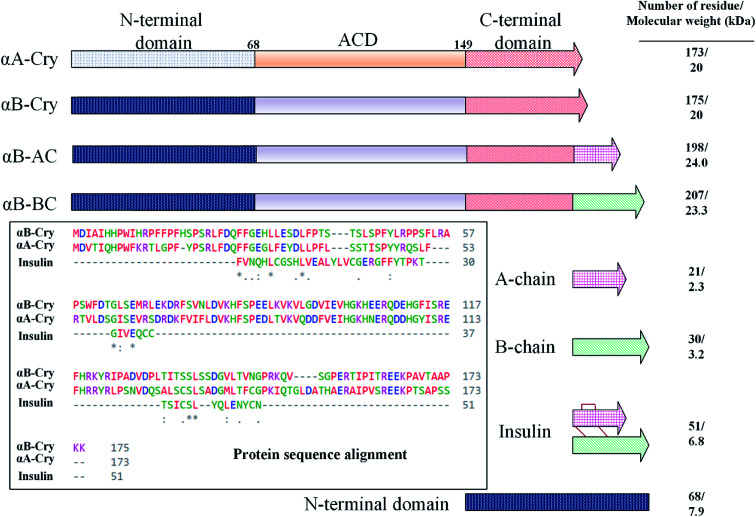
The schematic illustration of the seed-forming proteins and peptides. Selected eight protein and peptide models on the fibrillation of human insulin. As depicted, these models vary in their molecular masses and the number of residues. Human αA-Cry and αB-Cry share over 50% sequence similarity (multiple sequence alignment was done using Clustal Omega tool), also, both possess three structural regions – hydrophobic N-terminal domain (NTD), α-Cry domain (ACD) and C-terminal domain (CTD).

In this study, fibrillation kinetics of human recombinant insulin was examined in the presence of the fibril seeds from seven different proteins or peptides and the seed from insulin itself. Scheme 1 summarizes variation in the size, sequence, and hydrophobicity of the selected proteins or peptides.

**Fig. 1:**
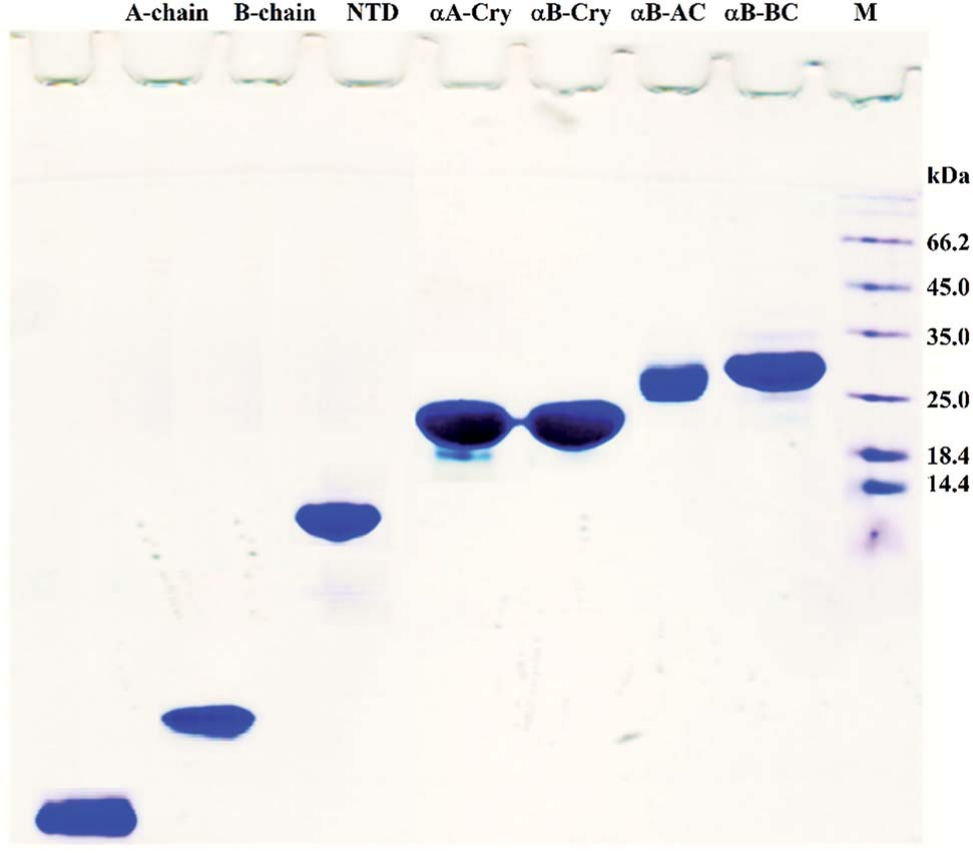
SDS-PAGE analyses for purification of the selected proteins and peptides. Human insulin A- and B-chain were purified by gel filtration chromatography. Human αB-Cry, human αA-Cry, human αB-BC, and human αB-AC were purified as described in published literature.^21^

The scheme exhibits intact human insulin and its A- and B-chain and each was studied separately. Next, αA- and αB-crystallins were included in this group. These two chaperone proteins have a mass of ∼20 kDa, share significant sequence identity and both possess three distinct structural regions; the hydrophobic N-terminal domain (NTD), α-crystallin domain (ACD) and hydrophilic C-terminal domain (CTD). The purpose of this selection was to investigate the impact, if any, of the sequence variation in αA- and αB-crystallins on human insulin fibrillation during cross nucleation. Additionally, the NTD fragment of human αB-crystallin (αB-NTD) was prepared with the aim to assess how seed of a hydrophobic protein will affect insulin fibrillation behavior. We also included a variant of human αB-Cry (M68I, P130V) that was individually fused to A- or B-chain of human insulin (αB-AC or αB-BC, respectively). Taken together, we prepared eight proteins and peptides, possessing the molecular masses of 24, 23.3, 20, 7, 5.8, 3.4, and 2.4 kDa, and their purity was checked by SDS-PAGE (Fig. 1). The fibril seeds were generated individually from all the above mentioned proteins and hybrid proteins (αB-AC and αB-BC) and were then used in secondary or cross nucleation to assess their impact on insulin fibrillation. The effect of nucleation on fibrillation kinetics, morphology, and oligomerization of insulin was characterized and compared.

### Primary structure plays an important role in fibrillation behavior of the model proteins and peptides

**Fig. 2:**
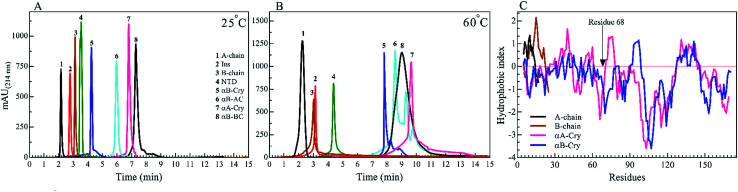
RP-HPLC analysis of the seed forming proteins and peptides at 25 °C and 60 °C. A 20 μL of each protein and peptides (2.0 mg mL^−1^) in the starting buffer (24% acetonitrile in water) was subjected to the column and run gradient over 15 min (1.0 mL min^−1^) using 24–60% acetonitrile in water. The hydrophobicity was analyzed by the Knauer HPLC system. The absorbance signals were recorded at 214 nm, using DAD 2.1 UV-Visible detector (Knauer, Germany). The experiments were conducted at 25 °C (A) and 60 °C (B). The hydrophobicity index *versus* amino acid residues is shown for insulin A-chain, insulin B-chain, human αA-Cry and human αB-Cry (C).

We started by studying the fibrillation of selected proteins by themselves. It has been suggested that protein sequences and hydrophobicity play an important role in the amyloidogenic nature of protein.^19,29^ Bis-ANS is a classical probe for the assessment of protein surface hydrophobicity^4,29,30^ but due to the controversial results with insulin and it's B-chain,^16^ it was not possible to accurately compare the bis-ANS-based measurement of surface hydrophobicity among our different seeding proteins and peptides. Therefore, to have a better picture of surface hydrophobicity of the selected proteins and peptides, they were applied to reverse-phase high-performance liquid chromatography (RP-HPLC) column at ambient temperature (Fig. 2A). In RP-HPLC, the retention of a protein of interest reflects its overall hydrophobicity; the more hydrophobic protein needs a longer time for the elution.^31^ As shown in Fig. 2A, A-chain with the least hydrophobicity among the model proteins and peptides was detected to elute as a peak at about 2.1 min, while αB-BC as the most hydrophobic protein eluted at 7.2 min. Based on our result, the surface hydrophobicity of these proteins and peptides is in the following order: αB-BC > αA-Cry > αB-AC > αB-Cry > αB-NTD > B-chain > insulin > A-chain. It should be noted that the fibril formation by these proteins and peptides was conducted at high temperatures (60 °C) so we also characterized surface hydrophobicity of these proteins and peptides at 60 °C (Fig. 2B). Interestingly, when they were studied at high temperature, the retention times for insulin, the A- and B-chain did not alter significantly, suggesting no important changes occurring in their surface hydrophobicity under thermal stress. The significant delay in the elution of αB-Cry and αB-AC hybrid protein suggests an important increment of their surface hydrophobicity upon thermal stress. The new elution peaks could be due to their intrinsic ability for oligomerization. Likewise, under this condition, the shifts of 1.0, 2.8 and 2.0 min were respectively realized for αB-NTD, αA-Cry and αB-BC proteins, suggesting the increment of their solvent-exposed hydrophobic surfaces or oligomerization state upon thermal stress. To gain an insight into the hydrophobic profile of the samples, the sequence hydrophobicity index of the samples was predicted using the parameters described by Kyte & Doolittle (Fig. 2C). In this type of prediction a value, GRAVY (grand average hydrophobicity), is associated with the hydrophobicity of proteins.^32^ High positive values suggest high hydrophobicity, whereas negative values indicate high solubility. Generally, regions in the N-terminal domain (residues 1–68) of αB- and αA-crystallins frequently exhibit positive GRAVY index, indicating these segments of the sequence are more hydrophobic than other segments in the proteins. In contrast, the 28 residues of the ACD have negative GRAVY values, indicating these areas are probably not hydrophobic. Remarkably, surface hydrophobicity assessments of A- and B-chain indicate high positive values. Although the theoretical data showed a significant difference in the hydrophobic nature of the samples, they were very different from the experimental point of view.

**Fig. 3:**
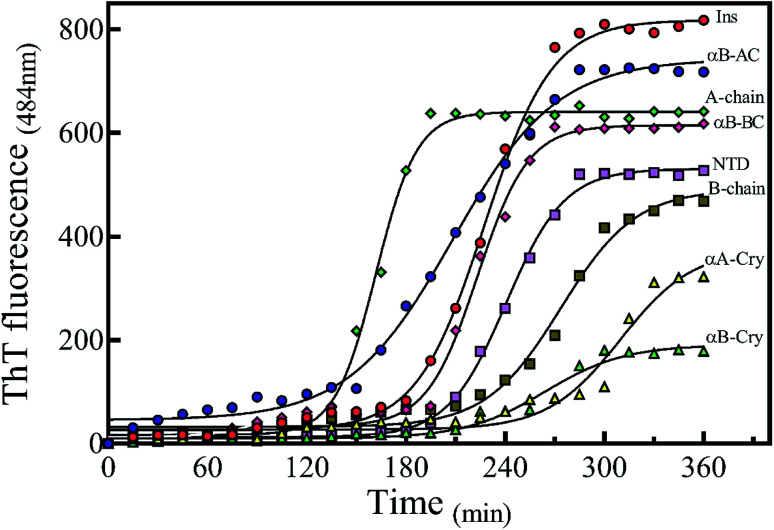
Monitoring the kinetics of protein and peptide models fibril formation by ThT fluorescence assessment. The fibril formation by different proteins and peptides was monitored by ThT fluorescence intensity. The concentration of each sample was fixed at 2.0 mg mL^−1^ with 10 μM ThT in 20% acetic acid at pH 2.0, containing 50 mM NaCl. The samples were incubated at 60 °C for 360 min to induce fibril formation. Also, at the desired time intervals, the analyses were conducted. The excitation wavelength of the protein/peptide samples was fixed at 450 nm, while the emission was recorded at 484 nm. The results were average of three independent experiments.

To analyze the kinetics of fibrillation and to prepare the seeds, these proteins and peptides were incubated under acidic conditions (pH 2.0) containing 50 mM NaCl at high temperature for 360 min. During incubation, the progress in fibril formation was monitored using a ThT-based assay (Fig. 3).

As shown in Fig. 3, ThT fluorescence emission intensities of A-chain and αB-AC increased rapidly, showing a lag time of lower than 140 min (Table 1). A-chain alone and A-chain fused to human αB-Cry revealed differences in the propensity for fibrillation under thermal stress. Comparing the fibrillation kinetics data of A-chain, αB-Cry and αB-AC, it can be concluded that insulin A-chain reinforces its strong fibrillation behavior on the fusion protein, although, A-chain forms about one-tenth of the primary structure of the fusion protein (αB-AC). The fibrillation propensity of the intact insulin molecules (lag time 185 min) was ranked significantly lower than A-chain alone but higher than B-chain by itself. Judging from these kinetic studies of ThT fluorescence assessment under thermal stress αB-NTD reveals significantly higher aggregation propensity than its parent molecule. The recombinant lens proteins αA-Cry and αB-Cry exhibited the least propensity for aggregation under thermal stress (Table 1). According to the data presented in Fig. 2B and 3, it is evident that poor aggregation of αA-Cry does not relate to the solvent-exposed hydrophobic surface indicated by this protein under similar thermal stress, suggesting the contribution of additional factors in fibrillation process of αA-Cry.

**Table 1:** Lag time and *T*_1/2_ of the proteins/peptides used in this study

	Lag time (min)	*T*_1/2_ (min)
αB-AC	131 ± 4.0	203 ± 4.0
A-chain	135 ± 3.0	163 ± 5.0
Insulin	146 ± 5.0	180 ± 4.0
αB-BC	187 ± 6.0	221 ± 6.0
αB-NTD	205 ± 8.0	240 ± 6.0
B-chain	220 ± 5.0	271 ± 8.0
αA-Cry	257 ± 7.0	303 ± 4.0
αB-Cry	268 ± 3.0	301 ± 5.0

**Fig. 4:**
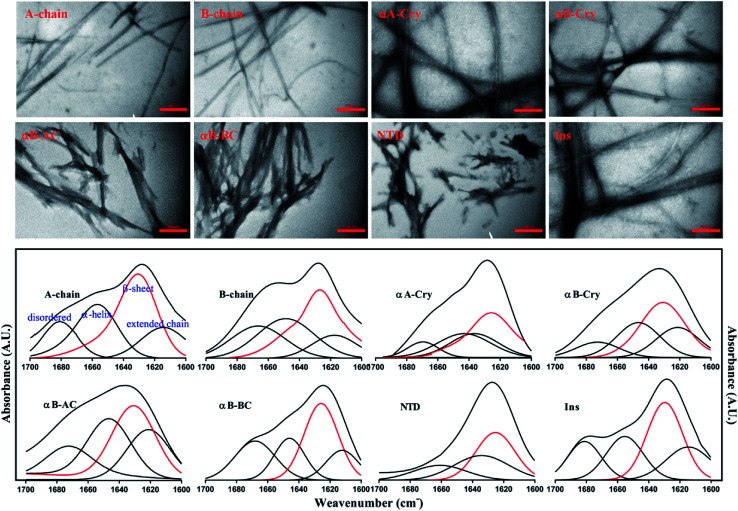
The morphological and structural assessments of the peptide and protein fibrils by TEM and FTIR spectroscopy. TEM (top panel) and FTIR spectroscopy analysis (down panel) of amyloid fibrils were carried out on the samples that were incubated for 6.0 h under the fibrillogenic conditions. All scale bars are 350 nm.

Next, the morphology and structure of the aggregates/fibrils were characterized by TEM imaging and FTIR spectroscopy (Fig. 4).

As shown in Fig. 4 (top panel), intact insulin, insulin B-chain, human αB-Cry and human αA-Cry formed the long straight fibrils while insulin A-chain, αB-AC, αB-BC and αB-NTD produced short fibrils that clumped with each other. The width of fibrils in all these conditions was between 20–45 nm. This morphology is similar to the amyloid or fibrillar aggregates reported for other proteins.^25,33,34^ In FTIR spectroscopy, the amide I region (1600–1700 cm^−1^) is obtained by backbone C=O and C–N stretching/vibration of the proteins and gives information about the secondary structure elements of the proteins.^23,35^ FTIR spectroscopy of the generated fibrils shows that fibrillation of all samples is accompanied by an increase in the β-sheet structure as indicated by a strong peak at 1628 cm^−1^ (ref. 36) in each sample. The other structural elements (*e.g.*, turns 1680 cm^−1^ and helical structure 1658 cm^−1^ were observed to populate in varying extents in each case). Interestingly, in the fibrils of αB-NTD, a peptide composed of 68 residues, there was no indication of the turn structures (absence of a peak at 1680 cm^−1^).^36,37^ Taken together, the ThT, TEM and FTIR data establish without doubt the production of fibrils from all the selected proteins under these conditions.

**Fig. 5:**
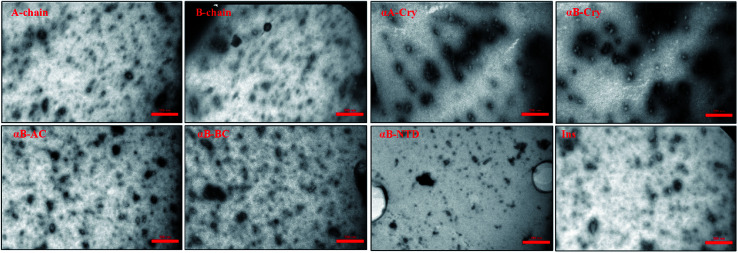
TEM images of the seeds. Seeds were prepared by sonication and then TEM imaging was conducted. The scale bares are 350 nm. The average length of all seeds was approximately 50 nm.

Mature fibrils of the investigated proteins and peptides were broken down by ultrasound waves using previously defined setup^26^ and TEM images of resulting mixtures were recorded (Fig. 5). These images show a uniform field of fibrillar aggregates in each picture. The average length of all seeds was approximately 50 nm. All of these seeds were used in the seeding and cross-seeding experiments as mentioned below.

### Only particular amyloid seeds are capable of enhancing the rate of insulin fibrillation

**Fig. 6:**
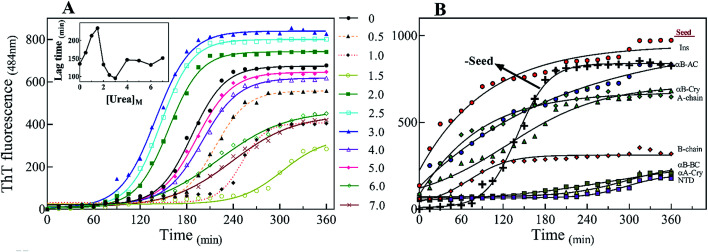
The impact of urea and seeding on the kinetics of insulin fibrillation. (A) Insulin fibril formation in the presence of different concentrations of urea. Insulin (2.0 mg mL^−1^) was incubated by urea in different concentrations (0.5–7.0 M). (B) Effect of seeding on insulin fibrillation. The seeds of different origins were added at 5% (W/W). The experiments were conducted under acidic conditions (20% acetic acid, pH 2.0) containing 50 mM NaCl at 60 °C. The plots are an average of three independent experiments.

There are abundant shreds of evidence in the literature indicating enhancement of protein fibrillation is generally induced through the generation of partially folded intermediates by environmental stresses.^38^ Given this assumption, it will be important to know if the seeds from different proteins and peptides enhance fibrillation especially in cross seeding cases and also if seeds induce any structural changes in monomeric insulin to catalyze the fibril conversion. Therefore, for reference, we used urea to induce structural alteration of human insulin and subsequently studied fibrillation kinetics of insulin in the presence of both an increasing concentration of urea (Fig. 6A) and in seeding/cross-seeding conditions (Fig. 6B).

The lag-phase for insulin in 0 M urea was 144 minutes. As urea concentration was increased stepwise to 1.5 M urea, a remarkable decrease in the lag time of insulin fibrillation (nucleation) was observed as indicating faster kinetics under these conditions (Table 2).

**Table 2:** Lag time and *T*_1/2_ of insulin fibrillation in the presence of different urea concentrations

Urea (Molar)	Lag time (min)	*T*_1/2_ (min)
0.0	144 ± 4.0	185 ± 5.0
0.5	170 ± 3.0	215 ± 4.0
1.0	213 ± 8.0	247 ± 3.0
1.5	235 ± 5.0	292 ± 6.0
2.0	110 ± 9.0	156 ± 7.0
2.5	102 ± 4.0	145 ± 6.0
3.0	93 ± 2.0	140 ± 8.0
4.0	149 ± 6.0	199 ± 7.0
5.0	138 ± 3.0	191 ± 3.0
6.0	130 ± 4.0	216 ± 4.0
7.0	154 ± 2.0	233 ± 5.0

Between 2.0 to 3.0 M urea the lag time decrease indicated an increase in fibrillation rate under these conditions. Above 3.0 M urea, the fibrillation rate of insulin again increased indicating a slowdown in the fibril formation under these conditions. Based on the results it appears that insulin in the presence of 0–2 M, 2–3 M, and >3 M urea adopts structurally distinct conformations, leading to a non-uniform propensity toward fibrillation. These experiments were followed by investigating the fibrillation of monomeric insulin in the presence of the protein and peptide seeds (Fig. 6B). The control of the human insulin monomer (symbol Fig. 6B) exhibited a lag time of 144 minutes. Secondary nucleation of insulin by the seed of insulin itself totally diminished the lag time of insulin fibrillation (red circle symbol Fig. 6B). Interestingly, the kinetics of fibrillation increased significantly by cross seeding of insulin itself by the seeds of αB-AC, αB-Cry, A-chain and B-chain although to different extents. On the other hand, seeds of αB-BC, αA-Cry and αB-NTD reduced fibrillation kinetics of human insulin.

**Fig. 7:**
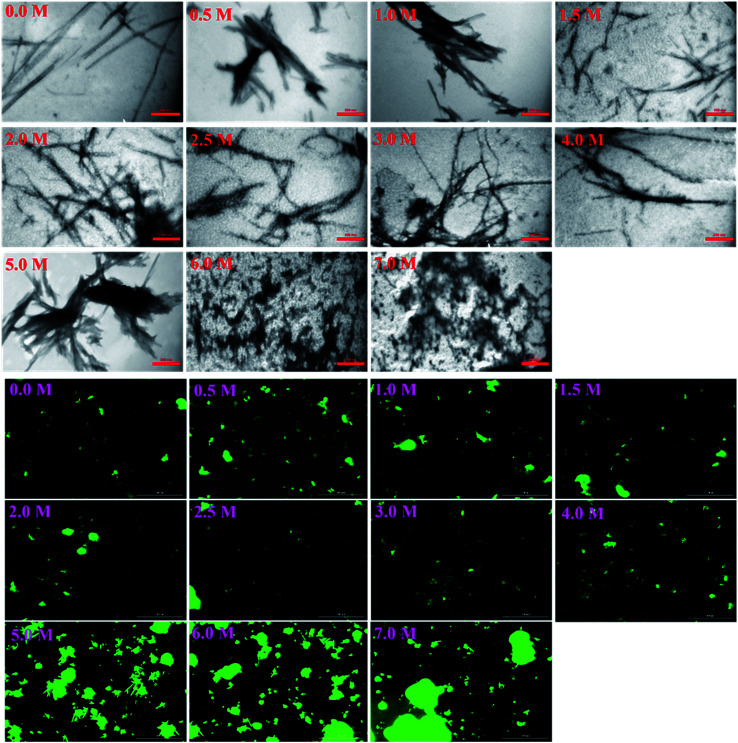
The TEM micrographs and fluorescence microscopic assessments of insulin in the presence of different concentrations of urea. Insulin fibrils were grown at pH 2.0 in different concentrations of urea. The scale bars for TEM micrograph and fluorescence images were 200 nm and 100 μm, respectively.

More details on the fibril morphology of human insulin under these stress conditions were inspected by TEM and fluorescence microscopy. Compared to insulin by itself, TEM images (Fig. 7, top panel) indicated fibrils that are generated in 2.0–3.0 M urea have straight and long morphology while the insulin fibrils generated in the 0.5–2.0 M urea revealed short and disordered and at times diffused shape. Further, by increasing urea concentration up to 4.0 M, the amorphous aggregates were observed along with minor fibrillar amounts. Highly aggregated monomeric insulin was seen in the presence of 6.0 and 7.0 M urea. Also, the zoom out imaging analyses of the insulin fibril populations by the microscopic fluorescence agreeably indicated comparable results (Fig. 7, down panel). Although the interaction between ThT molecules and amyloid β-sheets excites fluorescence property of the dye, some other studies have indicated the interactions between ThT dye and large amorphous aggregates by stacking in between the aggregates, leading to increased fluorescence.^39^

**Fig. 8:**
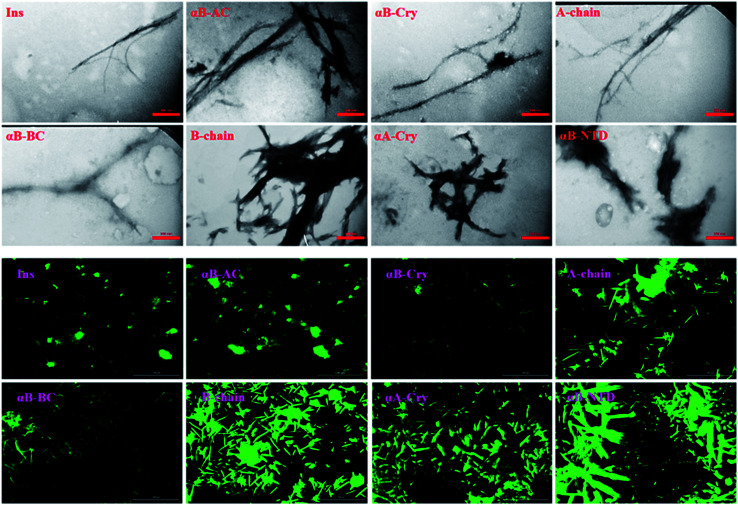
The electron micrograph and fluorescence images of insulin fibrils generated in the presence of different seeds. The images show the morphology of fibrils in the presence of different seeds at pH 2.0. The scale bars were 200 nm and 100 μm for EM and fluorescence images, respectively.

Likewise, the insulin fibrils made in the presence of the seeds were also examined by TEM and fluorescence microscopy. Judging from the results presented in Fig. 8, top panel, fibrils obtained in the presence of seeds of αB-AC, insulin, αB-Cry, A-chain, and αB-BC, possessed long, disperse and distinguishable morphology. However, seeds of insulin B-chain, αA-Cry and αB-NTD were observed to induce the formation of short and clumped fibrils of human insulin. Fluorescence microscopic images displayed comparable results, indicating separate and more notable fibrils of human insulin obtained from the seeds of αB-AC, insulin, αB-Cry, A-chain, and αB-BC in contrast to the short and clumped fibrils in other cases. Overall, our results suggested a specificity in fibril induction, while seeds of some proteins and peptides enhance the rate of insulin fibrillation others do not. Comparing Fig. 5 and 8, no evidence of residual seeds is apparent in the mixtures of the mature fibrils, indicating the investigated seeds were consumed in the fibrillation process.

**Fig. 9:**
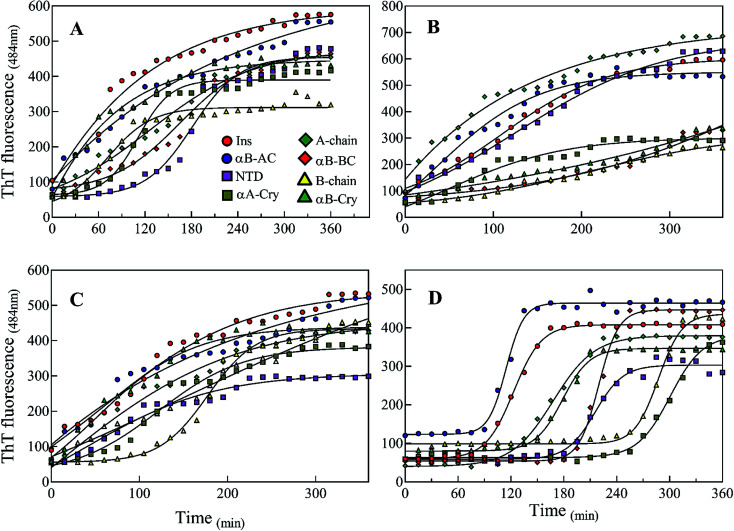
Kinetics of insulin fibrillation at pH 2.0 monitoring by ThT fluorescence assay. Co-incubation of the monomeric insulin (2 mg mL^−1^) with the seeds of different proteins and peptides (5% W/W) at 2.5 M (A) and 7 M urea (B). Also, before adding the seeds, insulin was pre-incubated at 2.5 M (C) and 7 M (D) urea and after that, the seeds were added to the mixtures.

Also, our data from urea-induced insulin fibrillation suggested that the kinetics of fibril formation in 2.0–3.0 M urea were faster than the kinetics of fibrillation in the other concentrations of this chaotropic agent (Fig. 6). Insulin appears to be in the fibrillation prone conformation in this range. In contrast, at concentration 7.0 M urea, insulin conformation is not very susceptible to amyloid fibrils (Fig. 6A and 7). Therefore, we wanted to measure the synergistic effects of the seeds and urea on the kinetics of insulin fibrillation under these two different scenarios. Seeds (5%) from different proteins were added to the insulin in the presence of 2.5 or 7.0 M urea and fibrillation monitored. The results are summarized in Fig. 9.

When compared to fibrillation of insulin by itself at 2.0 M urea concentration (lag time 110 min, Fig. 6A), in the co-presence of 2.5 M urea and seeds from different proteins, a significant enhancement (but to different degrees) in fibrillation kinetics was observed in all cases except for αB-NTD seeds (Fig. 9A). A similar enhancing effect was observed in the co-presence of 7 M urea and seeds from different proteins (Fig. 9B) when compared to 7.0 M urea (lag time 154 min, Fig. 6A) only. Analyzing Fig. 9A and B, we see the overall acceleration of fibrillation kinetics in the co-presence of urea and seeds but individual seeds did not exhibit the exactly comparable effect. These data indicate that urea at both concentrations alters insulin conformation so that it is highly prone to association with the seeds. We need to recall here that concerning control 0 M urea, urea at 2.0 M is accelerating and at 7.0 M is retarding the fibrillation kinetics of insulin but the presence of seeds always lead to increased fibrillation rate. These findings indicated that seeds might be capable to induce structural modifying effect on insulin under these conditions.

To realize the seed effect on insulin conformation, the monomeric insulin was pre-incubated with 2.5 or 7.0 M urea for 120 min, respectively. Based on the data in Fig. 9A, the mentioned time is sufficient for 2.5 and 7.0 M urea to induce partially unfolded conformations of human insulin.^40^ The seeds were added to the mixtures after the incubation and insulin fibril formation was monitored by ThT fluorescence intensity (Fig. 9C and D). For the set of samples where insulin was pre-incubated with 2.5 M urea (Fig. 9C), adding the seeds increased the rate of fibrillation except for B-chain seeds that exhibited a reduced rate. Interestingly, the set of samples where insulin pre-incubated with 7.0 M urea a dramatic effect was observed. Each seed had a conspicuous observable effect on the insulin fibrillation kinetics. While seeds form B-chain and αA-Cry reduced the fibrillation compared to insulin at 7.0 M urea (Fig. 6A), αB-AC, insulin, αB-Cry, A-chain, and αB-Cry exhibited fibrillation accelerating effect in decreasing order, respectively. αB-BC and αB-NTD showed similar kinetic in these stepwise sequential incubation experiments as compared to 7.0 M urea only. Taken together Fig. 9 thus establishes that seeds can induce conformational modification of insulin. In other words, when starting the conformation of insulin is more constrained as in Fig. 9D and after prolonged incubation in urea, the kinetic rate of fibrillation is slow making it difficult for seeds to recruit the conformationally relevant monomers onto their growing ends. But if the monomer structure is flexible (*e.g.*, at low urea concentration or co-addition) the seed attraction overcomes all other constrains and growing ends do not have to exert any additional structural modifications required to recruit the insulin monomers on the seed ends.

### CD analyses show both urea or seeds can induce the formation of insulin intermediate conformers

**Fig. 10:**
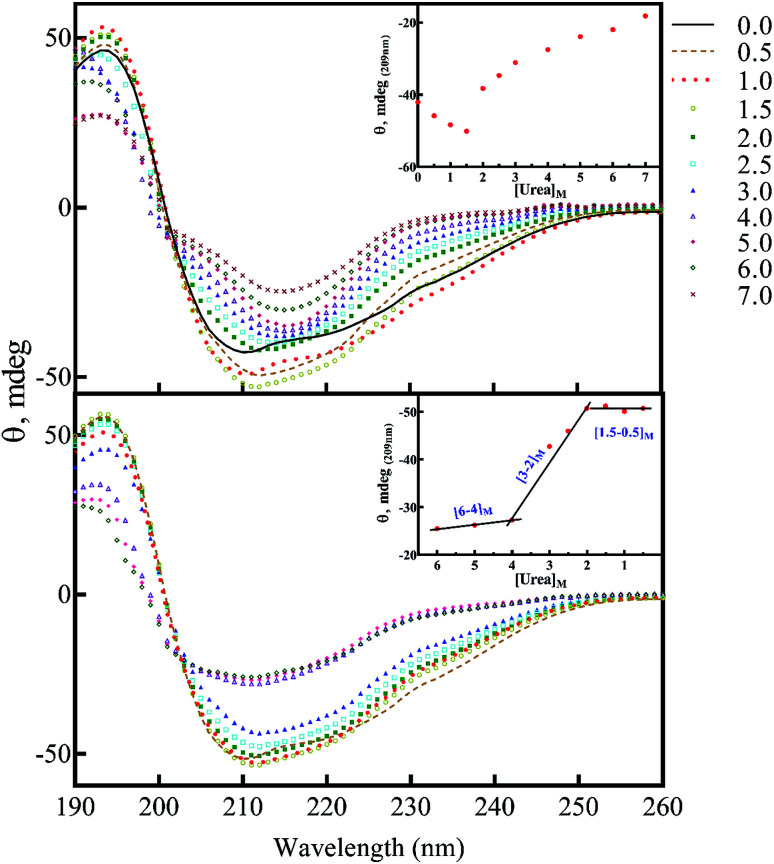
The secondary structural analyses of human insulin in the presence of different concentrations of urea. Human insulin (2.0 mg mL^−1^) was incubated (15 min) with different concentrations of urea (0.5–7.0 M), then the far UV-CD spectra were collected (A). The effect of decreasing concentration of urea on insulin secondary structure was also assessed (B). The dilution was carried out with a final insulin concentration of 2.0 mg mL^−1^ and different concentrations of urea. Before the dilution, insulin was incubated for 15 min. Then, upon dilution, the protein was incubated with the lower concentrations of urea for 15 min to equilibrate. The experiments were conducted at 60 °C, in 20% acetic acid, pH 2.0.

The above data, especially Fig. 9, motivated us to investigate the structural alterations of human insulin induced by different concentrations of urea and different protein/peptide seeds. The far-UV CD spectra (Fig. 10A) exhibited multifaceted behavior of insulin conformations in the range of 0–7.0 M urea concentration. An increase in the negative ellipticity at 222 nm indicated a large increase in the helical content of insulin between 0.5–1.5 M urea (Fig. 10A, inset) with the maximum helicity observed at 1.5 M urea. Increasing the concentration of urea from 2.0 to 7.0 M, a gradual decrease in the helical structures and an increase in beta and random coil structures were observed. It should be noted that these experiments were carried at 60 °C and β-sheet rich molecular association is possible. Overall the changes can be categorized in three phases; a structure inducing phase from 0.5–1.5 M, an unfolding phase (associated with oligomerization) from 2.0–7 M and a transition phase from 1.5 to 2 M urea. Next, we investigated whether the structural alterations of human insulin at the lower urea concentration are reversible. To do this, a fixed concentration of human insulin was incubated in different concentration of urea and then dilution to lower urea concentration was done in such a way that insulin concentration remains fixed at 2 mg mL^−1^ (Fig. 10B). As indicated, when urea was diluted from 7.0 to 4.0 M, no significant change in the unfolded insulin population was observed. However, reducing the urea levels in the range of 3.0 to 2.0 M, a remarkable recovery effect on the secondary structures of human insulin was observed. This finding indicates the dynamic and reversible fluctuation of the generated insulin intermediates in this range of urea concentrations. Finally, urea concentrations between 2.0–0.5 M did not have a significant effect on the secondary structure of insulin. These data when compared to fibrillation kinetics in Fig. 6 conclusively establish the contribution of the structural aspect of protein molecule in its fibrillation kinetics.

**Fig. 11:**
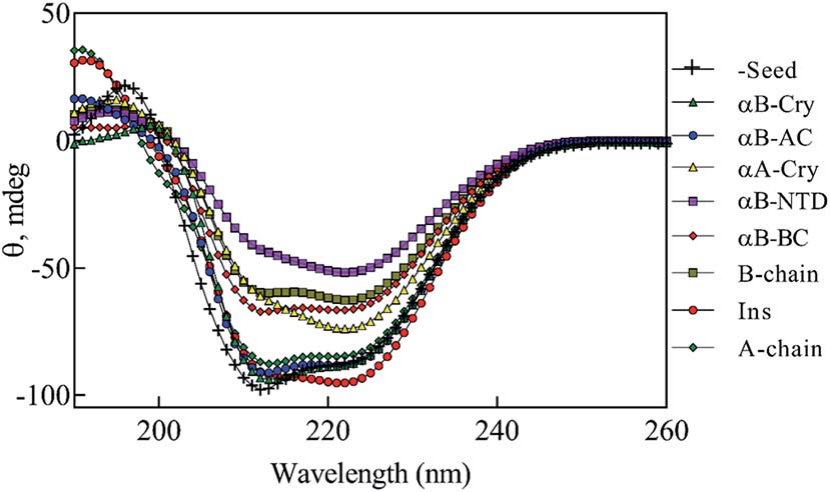
The seed-induced structural transition of human insulin. The far UV-CD analysis of human insulin (2.0 mg mL^−1^) in the presence of different seeds was done. Given 5% (w/w) seeds to insulin concentration, the final concentration of the seeds was 0.1 mg mL^−1^. Before collecting the spectra, insulin was incubated for 15 min in the presence of each seed. The experiments were conducted in 20% acetic acid containing 50 mM NaCl, 60 °C, at pH 2.0.

To study the seed-induced structural alteration of human insulin, the far-UV CD spectra of insulin were recorded in the presence of different seeds. Insulin was pre-incubated for 15 min in the presence of corresponding seeds at pH 2.0 (20% acetic acid, 60 °C) and then the CD data was collected. Insulin concentration was 2 mg mL^−1^ and the seed concentration was calculated to be 0.1 mg mL^−1^. Besides, the CD signal from each seed was subtracted from the final insulin CD spectra. Each CD profile in Fig. 11, therefore, represents the structure of insulin only.

As shown in Fig. 11, co-incubation of insulin by αB-NTD, B-chain, αB-BC and αA-Cry induced the structural transitions in human insulin, generating conformations which were similar to those changes observed in urea concentrations ranged from 4.0 to 7.0 M. Interestingly, the structural transitions induced by amyloid seeds of insulin, the A-chain, αB-AC and αB-Cry were comparable to those induced by 2.0–3.0 M urea. Although the dichotomy in this cohort of seeds in terms of structural modifications was evident in CD analysis their effect on fibrillation kinetics was much disperse.

If we summarily arrange the effect of various conditions on the *T*_1/2_ of the fibrillation in each condition (Fig. 11) we can perform a comparative analysis of the addition of each seed on insulin fibrillation. The analysis shows that insulin and αB-AC seeds are fibrillation inducers in all the cases whereas B-chain and αB-BC seeds either have no effect or decrease the fibrillation rate. The effect of A-chain and αA-Cry seed was observed to be indiscriminate. A-chain exhibited most random effect and varied from extreme inducer (7.0 M urea) to extreme inhibitor (2.5 M urea + seed) of the fibrillation and everything in between. αB-NTD showed an interesting overall effect. As seed itself or along with low concentrations of urea, αB-NTD inhibited the fibrillation of insulin as opposed to when the αB-NTD seeds along with urea or at high concentrations of urea only where αB-NTD induced the fibrillation. Fig. 11 also shows the order of proteins based on *T*_1/2_ values is different in the presence of seeds, urea, or in the absence of either indicating toward the contribution of conformational dispersion in different situations. It can also be observed that fibrillation pattern in the presence of seed and 2.5 M urea are significantly similar indicating convergence of underlying conformational states prone to fibrillation under these two different conditions. The summary suggests non-universality of seeding effect on fibrillation and the process is too complex to be categorized into an exclusively effect caused by hydrophobicity, size or origin of the seeds.

## Discussion

Accumulating evidence support cross-seeding of amyloid fibrillation. For example, the seeds of Aβ40, Aβ42, and αS have been reported to enhance the aggregation within themselves and among each other.^41^ Ren *et al.*^42^ have populated a list of different proteins and their counterparts exhibiting the cross-seeding behavior. Cross seeding has been termed “bidirectional” when two seeds from two proteins trigger each other's fibrillation (as in ref. 41); “unidirectional” (for example when Aβ seeds promoted hIAPP aggregation but hIAPP seeds did not for Aβ^18^); and “inhibitory” (when seeds from hIAPP derived peptides slowed down the fibrillation of insulin^11,43^). Further, two mechanisms have been proposed for cross-seeding; the ‘template-assisted’^15,44^ and ‘conformation selection and population shift’ mechanism. Cross-seeding has been suggested to exist in human diseases. For example, the association of Aβ and αSyn has been known for Lewy body dementia but relevant to our study in the pancreatic islet amyloid deposits of type-II diabetes patients Aβ, tau and hIAPP were found co-localized.^45^

**Fig. 12:**
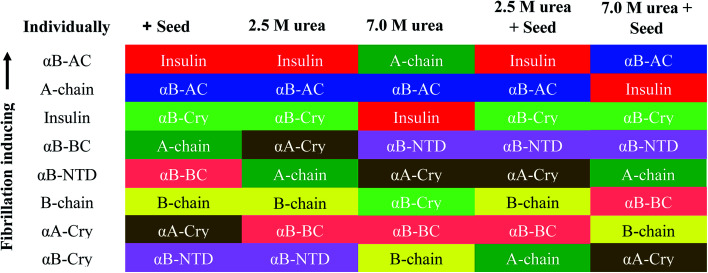
Comparative seeding effect on human insulin. The seeding effect was compared based on *T*_1/2_ value obtained in each condition and arranged in the decreasing order of *T*_1/2_ obtained in each seeding or cross seeding sample. The colors correspond to the seed used.

Given the above background, our studies provide different aspects of the fibrillation and cross-seeding induced fibrillation phenomenon. For example, based on several assumptions a popular model for amyloid fibrillation is based on the contribution of hydrophobic interaction among the partially unfolded states of the amyloidogenic proteins.^29,30,34^ Our results (Fig. 6–10) suggest this might not be the case and it is likely factors other than hydrophobicity may play an important role in altering the protein fibrillation kinetics. In this case, the human αA-Cry and αB-Cry studied here provide suitable examples. Although these proteins revealed a significant amount of solvent-exposed hydrophobic surfaces, they indicated a poor propensity for the thermal-induced fibrillation compared to the other proteins and peptides (Fig. 2 and 3). Specifically, these proteins indicated more hydrophobicity than insulin A- and B-chain at 25 °C and 60 °C yet their fibrillation lag time was meaningfully higher than that of insulin chains. Another aspect is the contribution of size. Our results in Fig. 1 and 3, suggested that the size of the proteins and peptides was not the determining factor associated with their susceptibility for fibrillation under these conditions. For example, the size of fusion proteins αB-AC (25.7 kDa) and αB-BC (26.7 kDa) is similar (4% difference) but the differences in the fibrillation propensity obtained from lag times (Table 1) are significant. αB-AC exhibited 30% faster fibrillation kinetics than αB-BC. While αB-AC indicated only 3% faster fibrillation compared to A-chain alone; αB-BC exhibited 15% faster fibrillation than B-chain alone. Between themselves, A-chain showed 42% faster kinetics than B-chain alone (at 30% size difference). Moreover, in the comparative seeding effect (Fig. 12) αB-AC exhibited increased fibrillation induction under all the cross-seeding conditions studied here but αB-BC was placed at the bottom with low cross-seeding fibrillation induction capacity. Our results indicated that size of the proteins has no significant effect on the kinetic of cross-seeding fibrillation. Perhaps, the primary structure can be considered to have the most remarkable influence on the fibrillation behavior of these proteins and peptides in conjunction with some previous studies.^5,13,19^ The marked increase in αB-fusion proteins compared to αB-Cry itself appears to fit this assumption well. Each constituent of the fusion proteins converts to fibrils individually so it cannot be ascertained from this data if the more aggressive aggregating constituent solely drives the faster kinetics of fusion protein or there is a more intricate mechanism involved.

Here we also want to clarify some choices that had to be adopted for technical reasons and feasibility of reactions. In the fusion forms of αB-Cry, the M68I mutation was made to prevent the formation of additional unwanted cleavage products in the presence of CNBr. The Asp-Pro sequence (residues 129 and 130) in αB-Cry is sensitive to the cleavage by formic acid and this reagent is needed for releasing of the insulin chains from the fusion proteins in the cleavage step. Therefore, Pro 130 was mutated to valine. Also, fusion forms of αB-Cry were used rather than αA-Cry because of the following reasons; the A- and B-chain of insulin has 3 and 2 cysteine residues, respectively. The αA-Cry has 2 cysteines in its sequence (residues 131 and 142). Due to the lack of cysteine in the sequence of αB-Cry it was considered a better choice for constructing the fusion protein to eliminate the effect of cysteine interaction in the structure. In the seed of fusion proteins, the accessibility of A- and B-chain for possible interactions with insulin is important.

**Scheme 2:**
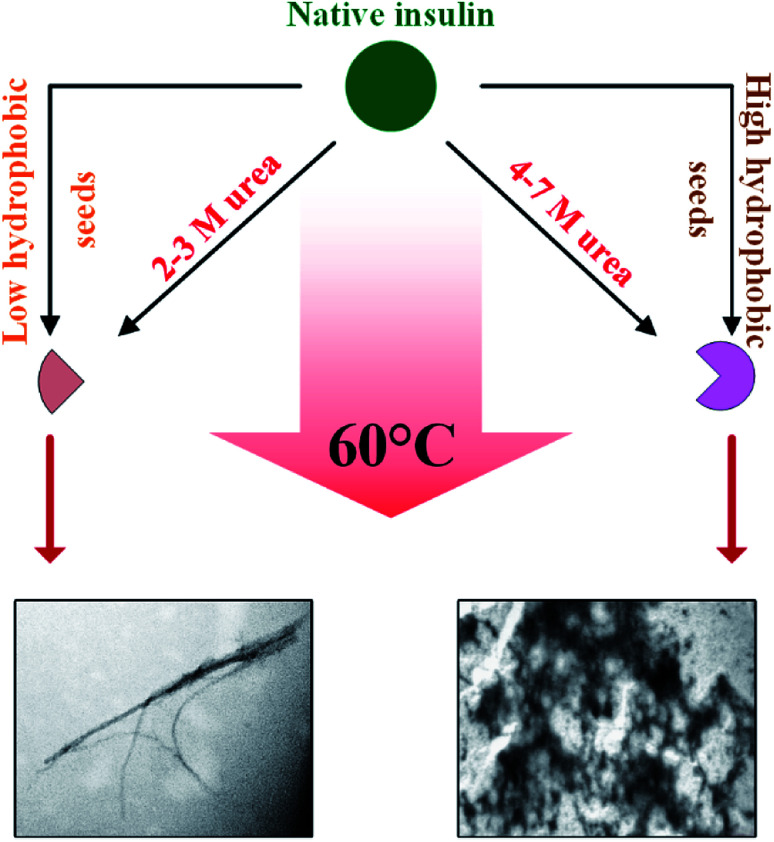
A proposed mechanism for the cross-templating insulin fibrillation. Native insulin in the presence of 2–3 M urea forms the intermediate conformers which subsequently produce ordered aggregates (amyloid fibrils), while urea at the higher concentration (4–7 M) induces the amorphous aggregation. The produced seeds with low hydrophobicity, similar to low urea level, are capable of inducing insulin intermediates-conformers that form long and distinguishable fibrils. Also, the seeds with high hydrophobicity, similar to high urea levels, were able to induce the entire unfolding of insulin, producing amorphous aggregates.

Focusing on the cross-seeding studies, so far, several efforts have been reported on studying the effect of the seed of one protein on fibrillation of another protein.^9,16,18,19,42^ In this context, we examined the possibility of cross-seeding of insulin amyloid formation with pre-formed amyloid seeds from many different proteins and peptides. We observed that the amyloid seeds of insulin, αB-AC, αB-Cry, A-chain and B-chain were capable of speeding up the kinetics of human insulin fibrillation (Fig. 9B). Also, our results indicated that seeds from αB-BC, αA-Cry, or αB-NTD significantly attenuated the fibrillation rate of human insulin. According to the results of our study, herein, we propose a new mechanism for the seed-induced insulin fibrillation. Insulin can be converted to various intermediates in the presence of different seeds. For example, Fig. 11 shows fibrillation inducing seeds like insulin, αB-AC or αB-Cry all have similar intensity at 209 and 224 nm but the αB-NTD or αA-Cry that have stronger intensities at 224 nm and correspondingly their seeds inhibit insulin fibrillation. Thus, some of the seeds with high molecular dynamics exhibit more susceptibility to form long and distinguishable fibrils. Although many different models for cross-seeding have been proposed^14,15,42,46^ our studies here provide the pivotal data in support of the template-assisted fibrillation model of cross-seeding.^42^ Further, we have thus different scenarios in the presence and absence of urea to compare. First, as human insulin cross-seeded by the low hydrophobic proteins and peptides, mimicking the narrow range of urea concentrations, the produced insulin conformers will be converted to the long fibrils. Second, non-fibrillar insulin aggregates will be formed in the presence of the seeds with high hydrophobic surfaces, mirroring the high urea levels (Scheme 2).

The results in Fig. 6, 7 and 9 suggested that those non-native insulin conformers which were produced in the presence of 2.0–3.0 M urea were much more susceptible to fibrillation. Besides, these intermediate species can recover their structures by reducing the urea concentrations, suggesting a high molecular dynamic propensity of the species.

Also, urea in the range of 4.0–7.0 M is capable of producing amorphous insulin aggregates (Fig. 7). Again, these data are in agreement with the previous studies^14,15,46^ the seeds of one protein can alter the conformational state of other proteins especially the template assembly (TA) models.^15^ In these two models, amyloid seeds act as a template to dictate a vulnerable state for soluble/folded proteins to fibrillate. Retrospectively, our data from RP-HPLC and far UV-CD studies (Fig. 2, 10 and 11) indicated that the seeds with a low degree of hydrophobicity (seeds of insulin, αB-AC, αB-Cry and A-chain) (Fig. 2) were capable to mimic the role of urea at the concentration range of 2–3 M, producing intermediate insulin conformers which were susceptible for the formation of long amyloid fibrils. However, the seeds of αB-BC, αA-Cry and αB-NTD with a higher level of hydrophobicity induce the entire unfolding of human insulin *via* hydrophobic interaction, resulting in the formation of amorphous aggregates under fibrillogenic condition. From these observations, if therefore is most likely that the insulin intermediate species induced by the urea and/or the amyloid seeds have a competitive relationship. Therefore, the core insulin molecule that contributes to fibril assembly is similar irrespective of being induced by the cross-seeding or denaturant.

Last but not least, it is worthwhile considering that the presented fibrillation condition was conducted at acidic pH (pH 2.0). At this pH, on account of pI of insulin (5.5) positive charges would be expected,^7^ likewise other involved in seed samples have positive charges. However, the interaction between insulin and preexisted seeds to form mature insulin fibril brings about the importance of hydrophobic interaction during seed-mediated fibril formation. When it comes to explaining the effects of electrostatic charges during the fibrillation, relay on our previous study,^10^ at physiological pH (7.4) the effect of seeding is expect to be similar as acidic pH due to negative charge of insulin and other seed-generated proteins (theoretically, pIs 6.82 and 5.84 were calculated for αB-Cry and αA-Cry, respectively).

Look over the study, we have focused on insulin which is an unstable protein with a high degree of conformational flexibility for extended periods in aqueous buffers.^47,48^ As a monomer, in the fibrillogenic condition (20% acetic acid), insulin may meet Leviathan's paradox, giving it too many accessible conformations to find a stable fold in a reasonable time frame.^49^ Here, through a combination of observations obtained from the seed-mediated and urea-induced insulin fibrillation, we have elucidated the similarities in the mechanism by which mild hydrophobic seeds of proteins and peptides are capable to provide the catalytic ends, inducing the formation of non-native insulin conformers.

## Conclusion

Through a combination of urea and seed-induced insulin fibrillation, we have identified a mechanism that monomeric insulin is efficiently incorporated into mature fibrils *via* cross-templating. These results indicate the core conformation of fibrillation prone intermediate is similar in either case. We also showed that the seeds of some proteins and peptides are capable to mimic the role of urea in inducing formation of the intermediate insulin conformation with the significantly increased rate of fibrillation kinetics. Finally, the seed-mediated insulin fibrillation conducted here will provide new insight into the specific role of seeds and the impact of cross-seed of different proteins and peptides in insulin fibrillogenesis.

## Conflicts of interest

The authors declare that they have no conflicts of interest with the contents of this article.

## Supplementary Material
